# Combining Metabolomics and Machine Learning to Identify Diagnostic and Prognostic Biomarkers in Patients with Non-Small Cell Lung Cancer Pre- and Post-Radiation Therapy

**DOI:** 10.3390/biom14080898

**Published:** 2024-07-24

**Authors:** Mauricio Murcia-Mejía, Marta Canela-Capdevila, Raquel García-Pablo, Andrea Jiménez-Franco, Juan Manuel Jiménez-Aguilar, Joan Badía, Rocío Benavides-Villarreal, Johana C. Acosta, Mónica Arguís, Alina-Iuliana Onoiu, Helena Castañé, Jordi Camps, Meritxell Arenas, Jorge Joven

**Affiliations:** 1 Department of Radiation Oncology, Hospital Universitari de Sant Joan, Institut d’Investigació Sanitària Pere Virgili, Universitat Rovira i Virgili, 43204 Reus, Spain; mauricio.murcia@salutsantjoan.cat (M.M.-M.); marta.canela@iispv.cat (M.C.-C.); raquel.garcia@iispv.cat (R.G.-P.); americadelrocio.benavides@salutsantjoan.cat (R.B.-V.); johana.acosta@salutsantjoan.cat (J.C.A.); monica.arguis@salutsantjoan.cat (M.A.); 2 Unitat de Recerca Biomèdica, Hospital Universitari de Sant Joan, Institut d’Investigació Sanitària Pere Virgili, Universitat Rovira i Virgili, 43204 Reus, Spain; andrea.jimenez@urv.cat (A.J.-F.); juanmaaguilar106@gmail.com (J.M.J.-A.); alinaiuliana.onoiu@urv.cat (A.-I.O.); helena.castane@iispv.cat (H.C.); jorge.joven@salutsantjoan.cat (J.J.); 3 Statistical Support Platform, Hospital Universitari de Sant Joan, Institut d’Investigació Sanitària Pere Virgili, Universitat Rovira i Virgili, 43204 Reus, Spain; joan.badia@iispv.cat

**Keywords:** biomarkers, lung cancer, metabolomics, radiation therapy, stereotactic body radiation therapy

## Abstract

Lung cancer is the leading cause of cancer-related deaths globally, with non-small cell lung cancer (NSCLC) accounting for over 85% of cases and poor prognosis in advanced stages. This study explored shifts in circulating metabolite levels in NSCLC patients versus healthy controls and examined the effects of conventionally fractionated radiation therapy (CFRT) and stereotactic body radiation therapy (SBRT). We enrolled 91 NSCLC patients (38 CFRT and 53 SBRT) and 40 healthy controls. Plasma metabolite levels were assessed using semi-targeted metabolomics, revealing 32 elevated and 18 reduced metabolites in patients. Key discriminatory metabolites included ethylmalonic acid, maltose, 3-phosphoglyceric acid, taurine, glutamic acid, glycocolic acid, and d-arabinose, with a combined Receiver Operating Characteristics curve indicating perfect discrimination between patients and controls. CFRT and SBRT affected different metabolites, but both changes suggested a partial normalization of energy and amino acid metabolism pathways. In conclusion, metabolomics identified distinct metabolic signatures in NSCLC patients with potential as diagnostic biomarkers. The differing metabolic responses to CFRT and SBRT reflect their unique therapeutic impacts, underscoring the utility of this technique in enhancing NSCLC diagnosis and treatment monitoring.

## 1. Introduction

Lung cancer stands as the predominant contributor to cancer-related fatalities worldwide, accounting for approximately 12% of all cases of cancer, with an annual incidence exceeding two million patients [1]. Non-small cell lung cancer (NSCLC) encompasses over 85% of lung cancer cases and is categorized into three principal histological subtypes: adenocarcinoma, squamous cell carcinoma, and large cell carcinoma. The five-year survival rate for early stage, operable NSCLC averages around 70%; however, it declines to 10–13% in advanced stages of the disease [2,3,4]. Presently, diagnosis heavily relies on symptomatology, often leading to late-stage detection and dismal prognoses. If the diagnosis could be shifted to early stages, the overall morbidity for this disease could profoundly decrease. Screening for lung cancer utilizing low-dose computed tomography has shown efficacy in mortality reduction [5]. However, the prevalence of individuals with indeterminate nodules, exorbitant costs, and resource constraints makes it necessary to investigate the discovery of more straightforward early diagnosis methods, ideally utilizing non-invasive or minimally invasive modalities, such as blood biomarkers [6], supplemented by clinical, epidemiological, imaging, and lifestyle data. This approach holds particular promise for individuals at high risk of lung cancer, as they may harbor subclinical disease for extended periods before symptomatic manifestation.

Recent research has underscored the pivotal role of investigating metabolic alterations in cancer development [7]. Cancer cell pathogenesis entails oncogene activation, apoptosis evasion, heightened replicative capacity, angiogenesis, and metabolic dysregulation, marked by alterations in signaling cascades, protein expression, and biochemical adaptation [8]. Predominant metabolic shifts in cancer cells encompass increased glucose consumption or aerobic glycolysis (the Warburg effect), augmented glutaminolysis, and heightened amino acid and lipid biosynthesis pathways [9,10,11]. Metabolomics emerges as a promising avenue in systems biology, aiming for the comprehensive interrogation of low-molecular-weight metabolites in biological specimens [12]. It is a potent tool for deciphering the biological pathways implicated in disease initiation and progression, furnishing invaluable insights into the molecular underpinnings of pathological processes [13]. Quantitative metabolomic profiling of plasma has been proposed as a candidate approach for lung cancer diagnosis and prognosis [14,15].

NSCLC patients typically undergo a treatment regimen comprising surgical resection, radiotherapy, chemotherapy, immunotherapy, and targeted therapy. In this study, we will refer to Conventionally Fractionated Radiation Therapy (CFRT) as encompassing two treatment schedules: normofractionated radiation therapy, which delivers 2 Gy per session on 30–33 consecutive fractions, and hypofractionated radiation therapy, which administers 4 Gy per session on 15 consecutive fractions. We will compare these treatments with another radiotherapy technique, stereotactic body radiation therapy (SBRT), also known as stereotactic ablative radiotherapy. SBRT has been introduced to improve treatment precision and reduce overall treatment time by delivering a narrow beam of high-dose radiation per fraction (>5 Gy to a specific target in fewer alternating fractions (between three and eight), ensuring accurate delivery to tumor tissue while sparing surrounding healthy tissue [16]. This treatment is commonly used for managing early stage tumors (less than 5 cm and with negative lymph nodes) in patients who are inoperable due to comorbidities, impaired respiratory function, or those who refuse surgery. In contrast, patients with inoperable locally advanced tumors (larger than 5 cm and with involved lymph nodes) are often treated with CFRT [17].

Therefore, this investigation aims to elucidate the shifts in circulating metabolite levels in NSCLC patients compared to healthy volunteers and delineate the effects induced by CFRT and SBRT. The overarching objective is identifying and proposing potential biomarkers for early diagnosis and prognosis of the disease.

## 2. Materials and Methods

We enrolled a cohort of 91 patients diagnosed with NSCLC who underwent either CFRT (38 patients) or SBRT (53 patients), collectively representing 99 treated lesions. All participants were treated at the Department of Radiation Oncology of the Hospital Universitari de Sant Joan de Reus between February 2013 and October 2022. Eligible patients exhibited a Karnofsky Performance Status Index > 70 and were classified as 0 or 1 on the Eastern Cooperative Oncology Group (ECOG) scale.

The radiotherapy protocols included normofractionated radiotherapy (total dose 54–70 Gy delivered at 2 Gy/day, five days/week), hypofractionated radiotherapy (total dose 60 Gy delivered at 4 Gy/day, five days/week), or risk-adapted SBRT (total dose 54–60 Gy, three alternated days/week) utilizing Volumetric Modulated Arc Therapy (VMAT) via the Varian RapidArc^®^ system (Varian Medical Systems, Palo Alto, CA, USA). Additionally, fourteen NSCLC patients received concurrent treatment with cisplatin (50 mg/m^2^) and etoposide (50 mg/m^2^) intravenously every three weeks.

Assessment of acute toxicity throughout the course of treatment was conducted weekly, employing the criteria established by the Radiation Therapy Oncology Group (RTOG) and the European Organization for Research and Treatment of Cancer (EORTC) [18].

Fasting blood samples were obtained at baseline and one-month post treatment. EDTA-plasma aliquots were promptly stored at −80 °C until metabolomic analyses were conducted. As a control group, we employed plasma samples from 40 healthy volunteers who had no clinical or analytical evidence of infectious disease, renal insufficiency, liver disease, neoplasia, or neurological disorders. These individuals were recruited from a population-based study conducted within our local region [19].

Semi-targeted metabolomics assessed the plasma levels of 74 metabolites involved in carbohydrates, amino acids, lipids, cofactors, vitamins, nucleotide pathways, energy metabolism, and xenobiotic biodegradation [20,21]. Plasma samples (50 μL) were mixed with an 8:2 (*v*/*v*) methanol: water solution containing internal standards, vortexed, and centrifuged. The supernatants (200 μL) were evaporated in a SpeedVac vacuum concentrator (Thermo Fisher Scientific, Waltham, MA, USA) and reconstituted with methoxyamine before silylation. Chromatographic separation was achieved using a 7890A gas chromatograph paired with a 7200-quadruple time-of-flight mass spectrometer equipped with an electron impact source (Agilent Technologies, Santa Clara, CA, USA). Moreover, the system was fitted with a 7693 autosampler module and a J&W Scientific HP-5MS column (30 ms 0.25 mm, 0.25 μm) from Agilent Technologies. Compound identification and semi-quantification were determined using Relative Units (RU) calculated based on compound area/internal standard area ratios, with ion selection guided by electron impact spectra and primary specific ions recorded in the Fiehn-pct-2013 spectral library.

Group comparisons were conducted using appropriate statistical tests based on variable types. Quantitative variables were analyzed by the Mann–Whitney U test (independent variables) or the Wilcoxon signed rank test (dependent variables), and results are shown as medians [interquartile ranges]. Categorical variables were evaluated by the χ² square test, and results are shown as number of cases (percentages). Significance was determined at *p* < 0.05. All analyses and plots were executed using RStudio version 4.3.1. Patient clinical characteristics were summarized using the TableOne package version 0.13.2. A logarithmic transformation was applied to metabolomic data. Orthogonal Partial Least Squares Discriminant Analysis (OPLS-DA) and Variable Importance in Projection (VIP) score analyses were performed using the ropls package version 1.32.0. Using the caret package version 6.0-94, a 5-fold cross-validated Support Vector Machine (SVM) multivariate model was trained on 70% of the data. The model was then tested on the remaining 30% of the data. Receiver Operating Characteristics (ROC) curves were generated using the pROC package version 1.18.5. Volcano plots and boxplots were created with the ggplot2 package version 3.5.1. All analyses utilized the latest package versions available on CRAN as of 28 April 2024.

## 3. Results

### 3.1. Clinical Characteristics of Study Participants and Their Tumors

While most participants were men, notable differences emerged between NSCLC patients and the control group. These variances encompassed age, comorbidities such as diabetes mellitus, arterial hypertension, and dyslipidemia, as well as lifestyle factors like smoking and alcohol consumption. Patients treated with SBRT had a higher proportion of women and a lower prevalence of smokers than those receiving CFRT. The predominant tumor types among NSCLC patients were adenocarcinoma and squamous cell carcinoma, with most lesions located in the right upper lobe, right lower lobe, or left upper lobe (Table 1).

### 3.2. Baseline Metabolite Levels Effectively Differentiate NSCLC Patients from the Control Group

**Figure 1:**
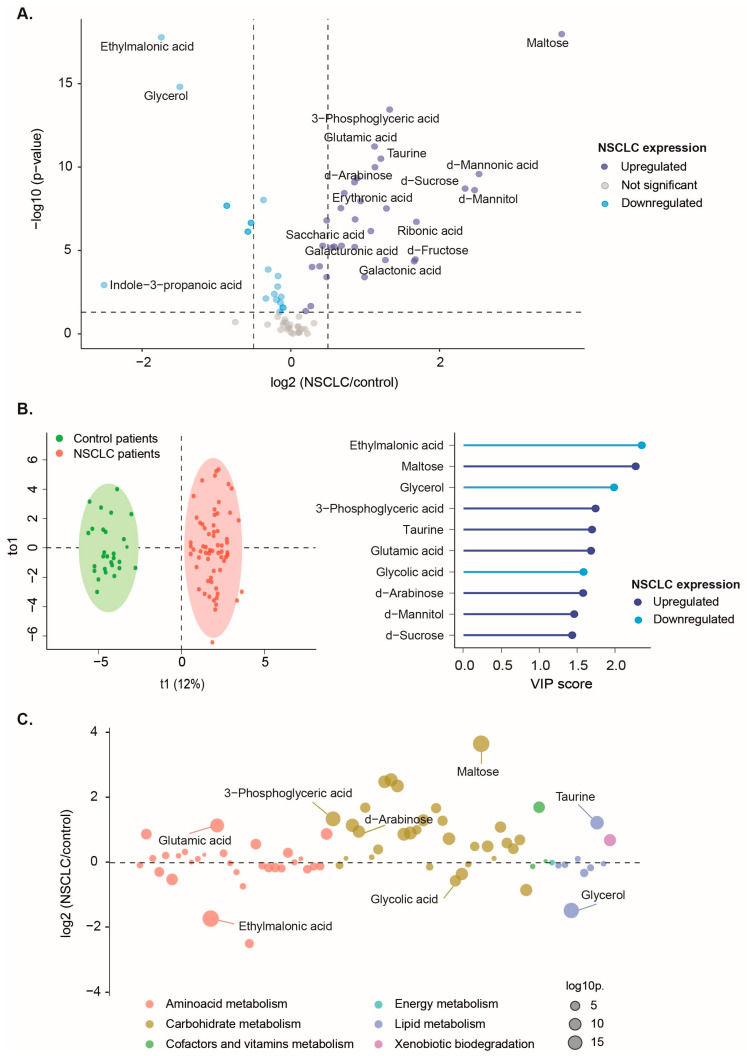
Comparative analysis of plasma metabolite profiles in non-small cell lung cancer (NSCLC) patients and the control group. (**A**) Differential expression of plasma metabolites revealed significantly upregulated and downregulated metabolites in patients compared to the control group. (**B**) Orthogonal Partial Least Squares Discriminant Analysis showed a clear distinction between the metabolic profiles of NSCLC patients and healthy individuals, with eight metabolites exhibiting a Variable Importance in Projection (VIP) score greater than 1.5. (**C**) The most significant metabolites distinguishing NSCLC patients from the control group were associated with amino acid, carbohydrate, and lipid metabolism.

A comparison of metabolite concentrations revealed 32 significantly higher and 18 lower plasma metabolite levels in NSCLC patients compared to the control group (Figure 1A and Supplementary Table S1).

OPLS-DA successfully distinguished the metabolic profiles of healthy individuals and NSCLC patients. Specific metabolites such as ethylmalonic acid, maltose, glycerol, 3-phosphoglyceric acid, taurine, glutamic acid, glycolic acid, and d-arabinose showed higher discriminatory capacity in segregating the groups, as indicated by their VIP scores exceeding 1.5 points (Figure 1B). Among these, ethylmalonic acid, glycerol, and glycolic acid plasma concentrations were lower in NSCLC patients. In contrast, maltose, 3-phosphoglyceric acid, taurine, glutamic acid, and d-arabinose were higher. These findings suggest potential metabolic alterations associated with NSCLC, particularly in amino acid, carbohydrate, and lipid metabolism (Figure 1C).

**Figure 2:**
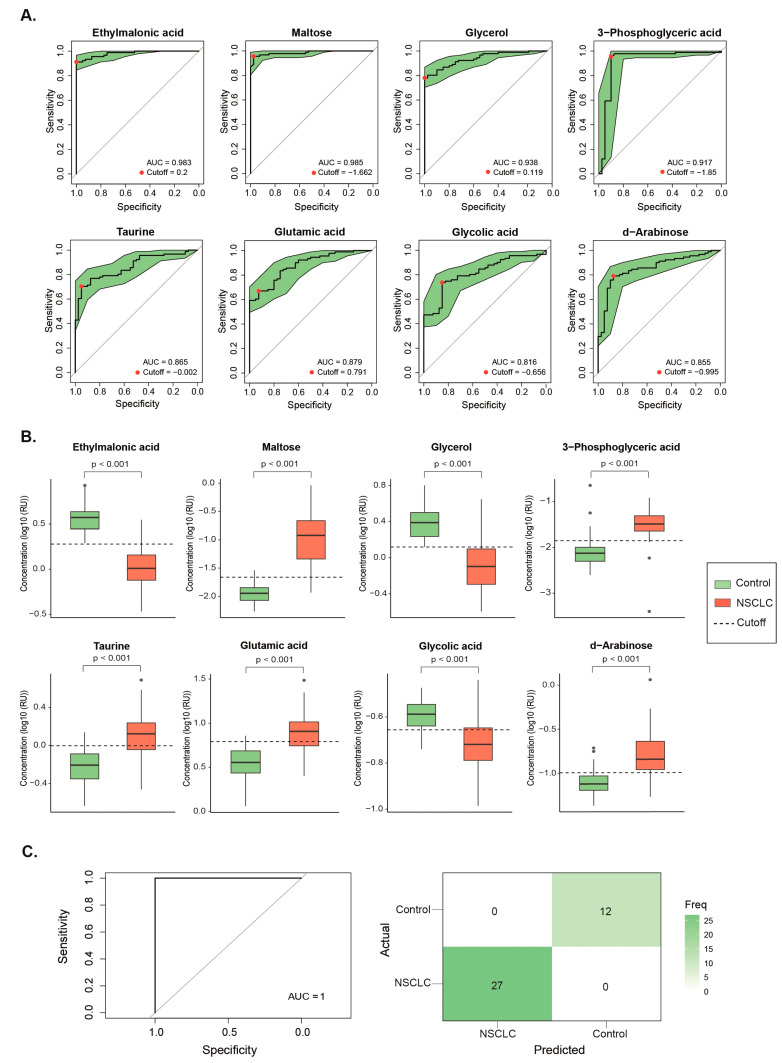
Plasma concentrations of ethylmalonic acid, maltose, glycerol, 3-phospogyceric acid, taurine, glutamic acid, glycolic acid, and d-arabinose had a high discriminating power to classify non-small cell lung cancer (NSCLC) patients from the control group. (**A**) Individual Receiver Operating Characteristics (ROC) curves showed an area under the curve (AUC) higher than 0.8. Red dots represent the optimal cutoff value determined by the Youden index for patient classification. (**B**) The Mann–Whitney U test revealed significant differences in plasma metabolite concentrations between NSCLC patients and healthy individuals. (**C**) The Support Vector Machine multivariate model showcased outstanding performance, with an ROC curve achieving an AUC of 1 and an unequivocal confusion matrix. Results and cutoffs are shown as log10 of Relative Units (RU).

For metabolites with VIP scores exceeding 1.5 points, we conducted univariate ROC curves, all of which yielded an area under the curve (AUC) higher than 0.8, indicating a high level of accuracy. Furthermore, the Youden index was computed for each curve to establish the optimal cutoff point for distinguishing NSCLC patients from healthy individuals (Figure 2A,B).

Finally, a SVM multivariate model was trained and tested based on those metabolites with a VIP score higher than 1.5 points. The ROC curve yielded an AUC of 0.999 for the training dataset (Figure S1) and 1 for the testing dataset (Figure 2C), indicating perfect discrimination between classes.

### 3.3. SBRT and CFRT Induce Distinct Changes in the Plasma Metabolome

**Figure 3:**
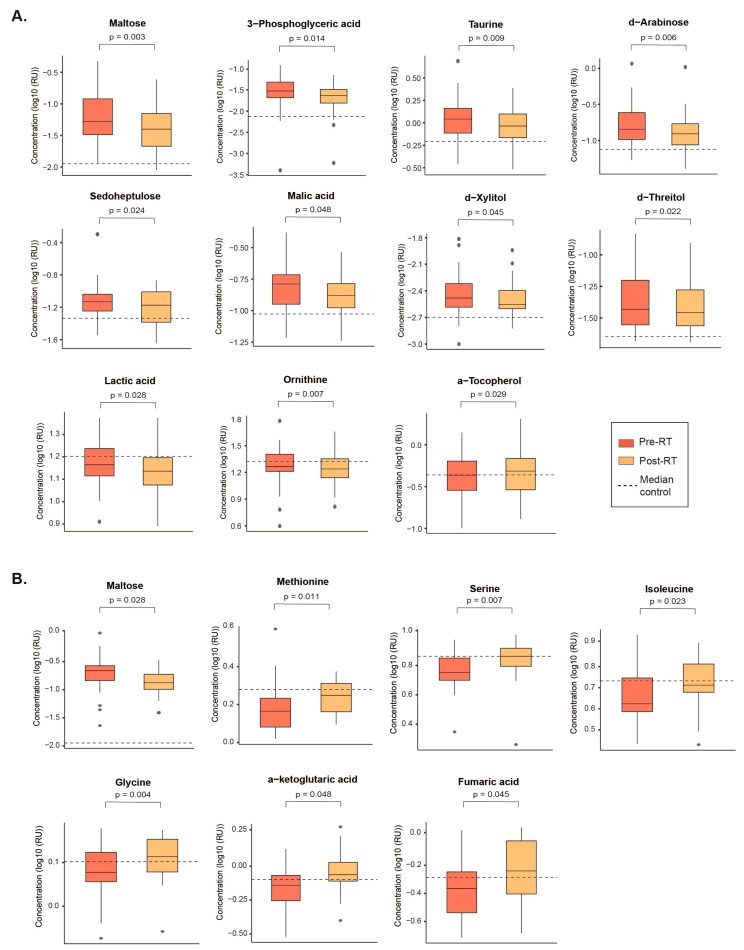
This figure illustrates metabolites that exhibited significant changes after treatment compared to pre-RT values with either (**A**) stereotactic body radiation therapy or (**B**) Conventional Fractionated Radiotherapy. Statistical significance was determined using the Wilcoxon signed-rank test. The dashed line indicates the median metabolite value for healthy volunteers. Results are presented as the log10 of Relative Units (RU).

The two radiation therapy modalities produced distinct changes in the metabolic profiles of NSCLC patients. SBRT led to decreased plasma concentrations of maltose, 3-phosphoglyceric acid, taurine, d-arabinose, sedoheptulose, malic acid, d-xylitol, and d-threitol, indicating a trend toward normalization. However, it also decreased levels of lactic acid and ornithine while increasing levels of α-tocopherol, further diverging from the profiles of healthy volunteers (Figure 3A and Supplementary Table S2).

In contrast, CFRT was associated with a lower concentration of maltose and higher concentrations of methionine, serine, isoleucine, glycine, α-ketoglutaric acid, and fumaric acid. These changes indicate a partial return to normal levels for these metabolites (Figure 3B and Supplementary Table S3).

It is noteworthy that maltose was the only metabolite exhibiting similar changes after both SBRT and CFRT.

## 4. Discussion

Our findings reveal a distinctive metabolic profile in individuals afflicted with NSCLC compared to healthy controls. The metabolites showing the most pronounced changes included maltose, 3-phosphoglyceric acid, taurine, glutamic acid, and d-arabinose, all of which had higher concentrations in NSCLC. Conversely, ethylmalonic acid, glycerol, and glycolic acid concentrations were decreased. The collective ROC curve generated from these metabolites effectively distinguished between patients and healthy controls, providing a clear and accurate classification.

Maltose, a disaccharide composed of two glucose molecules, arises from the breakdown of complex carbohydrates by the action of maltase. The higher plasma maltose concentrations found in NSCLC patients could be an adaptive response to the altered metabolic environment of the tumor, aiming to satisfy its energetic and biosynthetic needs [22].

3-phosphoglyceric acid is an intermediary metabolite in the glycolytic pathway that plays a crucial role in adenosine triphosphate generation. Under normal conditions, it converts into pyruvate, entering the mitochondria for complete oxidation in the tricarboxylic acid cycle. However, the persistence of heightened glycolytic activity in cancer cells, even in the presence of oxygen, is a hallmark of the Warburg effect [23]. Tumor metabolism prioritizes the generation of intermediates that support macromolecular biosynthesis, thereby providing a proliferative advantage. This metabolic shift likely leads to increased production of 3-phosphoglyceric acid; consequently, higher plasma concentrations in NSCLC patients can be found.

Taurine, also known as 2-aminoethanesulfonic acid, exhibits regulatory effects on various cellular processes, including the modulation of N-acetyl galactosaminyl transferase-2 expression and the downregulation of matrix metalloproteinase-2, thereby impeding invasion and metastasis [24]. Additionally, taurine demonstrates inhibitory effects on proliferation and pro-apoptotic properties in A549 lung cancer cells, with its efficacy being contingent upon dosage and exposure duration [25]. The increase in plasma concentrations of this amino acid in patients with NSCLC could be explained by a positive feedback mechanism attempting to counteract tumor development.

Glutamic acid assumes a pivotal role in metabolic pathways, with its involvement commencing at the onset of glutaminolysis, where glutamine undergoes conversion to glutamic acid catalyzed by glutaminase within the mitochondria. Studies have implicated glutamic acid as a discerning inflammatory marker in pulmonary pathologies [26]. Furthermore, in line with our results, consistent elevations in glutamic acid levels have been documented in patients with NSCLC, juxtaposed with a reduction in glutamine levels [27,28,29]. In addition, a significant increase in glutamic acid levels within lung cancer tissues compared to adjacent para-carcinomatous tissues has also been observed [30], suggesting a potential association between intratumoral glucogenesis and this amino acid.

The cell utilizes glycerol to synthesize intermediate metabolites essential for producing vital cellular components such as triglycerides and phospholipids. The observed lower concentrations of this metabolite in NSCLC patients may be attributed to its consumption by tumor tissue, where the accelerated synthesis of proteins, nucleic acids, and membranes occurs.

Insufficient data are available to fully interpret the underlying reasons behind the observed increase in d-arabinose concentrations and decreased glycolic acid and ethylmalonic plasma concentrations in NSCLC patients. D-arabinose is a five-carbon monosaccharide primarily found in specific plant sources, yet it is not commonly a part of human dietary intake. In humans, the endogenous synthesis of d-arabinose occurs in negligible quantities. Glycolic acid, a simple organic acid, is derived from the metabolism of lactic acid catalyzed by lactate dehydrogenase. However, its role in central metabolic pathways is relatively limited compared to other compounds, and it does not play a pivotal role in any significant metabolic pathways [31]. The decline in glycolic acid levels of LC patients might be attributed to its utilization as an energy source. On the other hand, ethylmalonic acid is a branched-chain fatty acid that has garnered attention in investigating neurological diseases associated with inborn metabolic disorders. Interestingly, patients with inborn errors of metabolism are predisposed to developing cancer [32]. However, the potential role of ethylmalonic acid in the pathophysiology of cancer onset and development has not been reported to date.

The observed metabolic profile in individuals with NSCLC indicates potential metabolic adaptations associated with cancer progression. Elevated concentrations of maltose, 3-phosphoglyceric acid, taurine, glutamic acid, and d-arabinose suggest increased glycolytic activity, potential regulatory effects on cellular processes, and alterations in glutamine metabolism, which are consistent with metabolic reprogramming observed in cancer cells. Conversely, decreased levels of ethylmalonic acid, glycerol, and glycolic acid may reflect altered utilization of these metabolites in cancer cells for biosynthesis and energy production.

In recent years, some studies have explored metabolic changes in patients with lung cancer, aiming to utilize these alterations for improved disease diagnosis and prognosis. These investigations have identified potential biomarkers to distinguish between patients and healthy individuals and differentiate among various types and stages of the disease. For instance, Qi et al. [33] identified palmitic acid, heptadecanoic acid, 4-oxoproline, tridecanoic acid, and ornithine as potential candidates for lung cancer screening, achieving a fair discriminative ability with AUC values ranging from 0.82 to 0.86. Similarly, Zhang et al. [34] highlighted the predictive potential of phenylalanine, phosphoethanolamine, xanthosine, dehydroepiandrosterone, glucose, fructose, and phenylacetic, hydroxyoxoglutaric, allocolic, aminopentanoic, and uric acids in assessing the stage and progression of lung cancer, exhibiting moderate discrimination with AUC values between 0.70 and 0.80. More promising results were reported by Zhang et al. [35], with the identification of lysophosphatidylcholine 20:3, phosphatidylcholine 40:6, citric acid, hydroxybutyric acid, and fumaric acid showcasing a diagnostic accuracy of 0.90 for early stage NSCLC. Furthermore, a previous study from our group [36] demonstrated that glutamic acid determination alone could diagnose lung cancer with an accuracy of 0.90. Remarkably, the findings presented in our present article underscore the exceptional discriminatory capacity of our identified panel, achieving an AUC of 1.0 and demonstrating perfect discrimination between cases and controls. This development holds great promise for the future of NSCLC diagnosis.

The present study introduces a novel aspect by identifying the metabolic changes induced by CFRT and SBRT in patients with NSCLC. Both modalities tend to normalize the concentrations of some metabolites while altering others, with the affected metabolites differing between treatments. The differences in metabolic effects between CFRT and SBRT reflect the distinct biological responses to these therapies. Understanding these alterations requires considering several factors, including the mechanisms of action, the biological pathways involved, and the intensity and precision of the radiation delivered.

CFRT brought about a partial normalization of plasma levels of maltose, methionine, serine, and isoleucine while elevating the concentrations of glycine, fumaric, and α-ketoglutaric acids beyond the median levels observed in healthy controls. Partial normalization of circulating levels of maltose and amino acids such as methionine, serine, and isoleucine after CFRT may reflect a decrease in carbohydrate metabolism and protein synthesis due to tumor mass reduction [36,37]. This observation suggests that CFRT might help restore normal cellular functions or reduce tumor-related disruptions in these pathways. On the other hand, increases in fumaric acid and α-ketoglutaric acid may indicate heightened oxidative stress and metabolic activity in response to radiation. These metabolites are tricarboxylic acid cycle components, suggesting enhanced mitochondrial activity or a stress response [38,39].

In contrast, SBRT significantly reduced the plasma concentrations of maltose3-phosphoglyceric acid, taurine, d-arabinose, sedoheptulose, malic acid, d-xylitol, and d-threitol toward normalization. Furthermore, SBRT induced decreases in lactic acid and ornithine concentrations, as well as an increase in α-tocopherol, thereby further distinguishing the metabolite profiles of LC patients from those of healthy controls.

The reduction of circulating levels of maltose, 3-phosphoglyceric acid, taurine, sedoheptulose, malic acid, and ornithine produced by SBRT could indicate a restoration of critical metabolic pathways such as the tricarboxylic acid cycle, the pentose cycle, and amino acid metabolism [40,41,42,43]. Changes in the levels of molecules generally foreign to the human body, such as d-arabinose, d-xylitol, and d-threitol, are challenging to interpret. However, one could speculate that they indicate alterations in the microbiome [8,44]. The decrease in lactic acid and ornithine may suggest a reduction in aerobic glycolysis (Warburg effect) and altered amino acid metabolism. Indeed, lactic acid is a byproduct of glycolysis, commonly elevated in tumors due to rapid cell proliferation [45]. A decrease in lactic acid suggests more efficient targeting of tumor cells and possibly better oxygenation of tissues post-treatment. Furthermore, an increase in α-tocopherol suggests an enhancement of antioxidant defenses. α-Tocopherol is a potent antioxidant, and its increase might be a protective response to mitigate radiation-induced oxidative damage [46].

The metabolic effects of CFRT and SBRT may differ significantly due to the distinct characteristics of each treatment and the cancer stage they target. CFRT administers lower doses of radiation per fraction over an extended period, causing sustained but less intense metabolic stress. This approach is typically applied to inoperable locally advanced tumors larger than 5 cm with involved lymph nodes. As a result, it may lead to gradual metabolic normalization and a steady stress response. In contrast, SBRT targets early stage tumors smaller than 5 cm with negative lymph nodes, delivering higher doses in fewer fractions, which results in acute and intense metabolic stress [47]. Moreover, CFRT affects a broader area, including surrounding healthy tissue, leading to extensive metabolic alterations as both tumor and normal cells respond to the radiation. Conversely, the high precision of SBRT targets the tumor more accurately, sparing healthy tissue and focusing the metabolic changes on tumor cell death, thereby reducing systemic metabolic disruption. In addition, SBRT activates the immune system more intensely than CFRT, potentially underlying the abscopal effect, which refers to effects observed at sites distant from the radiation administration [48,49,50]. This phenomenon presents a new avenue of research on the impact of these therapies on the immunometabolism of patients with lung cancer [51].

This study has its limitations. The sample size and demographics of the population studied may not fully represent the broader population affected by NSCLC, potentially limiting the generalizability of the findings. Variations in cancer stage among patients treated with SBRT or CFRT complicate attributing the observed metabolic changes solely to the type of radiotherapy administered. The cross-sectional design also hinders the establishment of causality or assessment of temporal relationships between biomarker levels and disease progression. Furthermore, interpreting metabolic changes in tumor tissue based on the measurement of circulating metabolites remains challenging. Addressing these limitations through more extensive multicenter, prospective studies, leveraging the vast amount of information provided by multi-omics approaches and artificial intelligence, could enable radiotherapy dose adjustments based on biomarkers related to tumor characteristics and radioresistance, thereby significantly improving the accuracy of NSCLC diagnosis and prognosis.

## 5. Conclusions

Our study identified significant metabolic alterations in NSCLC patients compared to healthy controls, highlighting specific metabolites which exhibited potential as biomarkers for early diagnosis. Both CFRT and SBRT induced distinct changes in the plasma metabolome, reflecting different metabolic responses to these treatments. Our findings underscore the potential of metabolomic profiling in improving lung cancer diagnosis, monitoring therapeutic responses, and guiding personalized treatment strategies.

## Figures and Tables

**Table 1:** Main characteristics of lung cancer patients, their tumors, and the control group.

	Control Group	All NSCLC Patients	CFRT	SBRT	*p*-Value
Patient characteristics	*n* = 40	*n* = 91	*n* = 38	*n* = 53	
Age (years)	69.50 [65.0–74.0]	73.00 [66.5–79.0]	71.50 [66.8–77.8]	74.00 [67.0–79.0]	*
Women	12 (30.0)	39 (42.9)	8 (21.1)	31 (58.5)	#
Smoking habit	9 (22.5)	69 (76.7)	34 (91.9)	35 (66.0)	* #
Alcohol habit	20 (50.0)	20 (22.0)	11 (28.9)	9 (17.0)	*
Diabetes mellitus	3 (7.5)	27 (30.0)	11 (29.7)	16 (30.2)	*
Arterial hypertension	13 (32.5)	56 (62.2)	21 (56.8)	35 (66.0)	*
Dyslipidemia	6 (15.0)	43 (47.3)	19 (50.0)	24 (45.3)	*
Non-cancer pulmonary disease	NA	58 (63.7)	31 (81.6)	27 (50.9)	NS
Cardiovascular disease	NA	34 (37.4)	20 (52.6)	14 (26.4)	#
**Cancer characteristics**		***n* = 99**	***n* = 38**	***n* = 61**	
Histology					NS
Adenocarcinoma	NA	44 (44.4)	15 (39.5)	29 (47.5)	
Squamous cell carcinoma	NA	42 (42.4)	21 (55.3)	21 (34.4)	
Others	NA	8 (8.1)	2 (5.3)	6 (9.8)	
Not determined	NA	5 (5.1)	-	5 (8.2)	
Stage					#
Ia	NA	60 (60.6)	13 (34.2)	47 (77.0)	
Ib	NA	15 (15.2)	6 (15.8)	9 (14.8)	
IIa	NA	1 (1.0)	1 (2.6)	0 (0.0)	
IIb	NA	8 (8.1)	3 (7.9)	5 (8.2)	
IIIa	NA	5 (5.1)	5 (13.2)	0 (0.0)	
IIIb	NA	8 (8.1)	8 (21.1)	0 (0.0)	
IIIc	NA	1 (1.0)	1 (2.6)	0 (0.0)	
IV	NA	1 (1.0)	1 (2.6)	0 (0.0)	
Tumor location					NS
RUL	NA	27 (27.6)	10 (26.3)	17 (28.3)	
RML	NA	5 (5.1)	2 (5.3)	3 (5.0)	
RLL	NA	21 (21.4)	11 (28.9)	10 (16.7)	
LUL	NA	27 (27.6)	11 (28.9)	16 (26.7)	
LLL	NA	18 (18.4)	4 (10.5)	14 (23.3)	

* *p* < 0.05, cancer vs. control group; # *p* < 0.05, SBRT vs. CFRT. Age is shown as medians [interquartile ranges], and differences were analyzed by the Mann–Whitney U test. Qualitative variables are shown as n (%), and differences were analyzed by the Χ^2^ test. CFRT: Conventionally Fractionated Radiation Therapy; LLL: left lower lobule; LUL: left upper lobule; NA: not applicable; NS: not significant; NSCLC: non-small cell lung cancer; RLL: right lower lobule; RML: right middle lobule; RUL: right upper lobule; SBRT: stereotactic body radiation therapy.

## Data Availability

The raw data supporting the conclusions of this article will be made available by the authors on request.

## Supplementary Materials

The following supporting information can be downloaded at: https://www.mdpi.com/article/10.3390/biom14080898/s1, Table S1: Plasma metabolite profiles of the control group and non-small cell lung cancer (NSCLC) patients pre-radiation therapy; Figure S1: Receiver Operating Characteristic (ROC) curve and confusion matrix generated using the training set of the data and a 5-fold cross validated Support Vector Machine model based on the 8 selected metabolites. The model distinguishes between non-small cell lung cancer patients (NSCLC) and the control group; Table S2: Plasma metabolite profiles of non-small cell lung cancer (NSCLC) patients pre- and post- SBRT); Table S3: Plasma metabolite profiles of non-small cell lung cancer (NSCLC) patients pre- and post- CFRT).
