# Supplementary material for: Combining Metabolomics and Machine Learning to Identify Diagnostic and Prognostic Biomarkers in Patients with Non-Small Cell Lung Cancer Pre- and Post-Radiation Therapy
Source: Biomolecules. 2024 Jul 24;14(8):898. doi: 10.3390/biom14080898 (PMC11353221; doi:10.3390/biom14080898)
Supplement: Supplementary file 1 [file biomolecules-14-00898-s001.zip › biomolecules-3082466-supplementary.pdf]

Table S1. Plasma metabolite profiles of the control group and non-small cell lung cancer (NSCLC) patients pre-radiation therapy.

Plasma metabolites	Control group<br>*n* = 40	All NSCLC patients<br>*n* = 91	p-value
Pyruvic acid	6.11 [5.06-7.46]	6.40 [4.72-8.46]	0.725
Lactic acid	15.93 [13.27-17.39]	14.28 [12.56-16.91]	0.202
2-Hydroxyisobutyric acid	0.33 [0.27-0.42]	0.44 [0.35-0.65]	**<0.001**
Glycolic acid	0.26 [0.23-0.28]	0.19 [0.16-0.23]	**<0.001**
Alanine	2.80 [2.34-3.49]	2.91 [2.53-3.49]	0.357
2-Hydroxybutyric acid	4.38 [3.60-5.76]	4.02 [2.86-5.58]	0.205
3-methyl-2-oxobutyric acid	0.17 [0.13-0.20]	0.11 [0.09-0.15]	**<0.001**
3-hydroxybutyric acid/3-hydroxyisobutyric acid	1.88 [1.35-2.67]	1.59 [0.89-4.44]	0.518
2-Hydroxyisovaleric acid	1.29 [1.03-1.64]	1.50 [1.08-2.74]	0.093
2-keto-3-methylvaleric acid	0.30 [0.23-0.34]	0.22 [0.17-0.28]	**<0.001**
3-Hydroxyisovaleric acid	0.25 [0.21-0.30]	0.30 [0.21-0.36]	**0.043**
Valine	18.91 [17.34-20.69]	17.29 [14.90-20.19]	**0.006**
Benzoic acid	0.26 [0.18-0.35]	0.25 [0.20-0.34]	0.974
Ethanolamine	0.33 [0.28-0.38]	0.29 [0.25-0.36]	0.136
Leucine	10.00 [9.32-11.28]	8.99 [7.23-10.85]	**0.001**
Phosphoric acid	3.83 [2.99-4.83]	3.73 [2.88-4.39]	0.449
Glycerol	2.44 [1.72-3.16]	0.80 [0.51-1.25]	**<0.001**
Ethylmalonic acid	3.74 [2.79-4.33]	1.02 [0.75-1.44]	**<0.001**
Isoleucine	5.40 [4.79-6.01]	4.70 [3.95-5.85]	**0.027**
Proline	20.83 [17.32-28.22]	22.19 [14.21-30.23]	0.863
Glycine	1.26 [1.20-1.36]	1.26 [1.15-1.36]	0.405
Succinic acid	0.08 [0.07-0.09]	0.10 [0.09-0.13]	**<0.001**
Glyceric acid	0.58 [0.52-0.74]	0.70 [0.56-0.93]	**0.022**
Fumaric acid	0.52 [0.38-0.74]	0.42 [0.25-0.61]	**0.049**
Serine	7.30 [5.90-7.96]	5.87 [4.89-7.20]	**0.004**
Threonine	7.68 [6.56-8.83]	6.71 [5.71-8.01]	**0.014**
Hydrocinnamic acid	0.15 [0.08-0.23]	0.11 [0.07-0.18]	0.196
Malic acid	0.09 [0.08-0.12]	0.14 [0.10-0.19]	**<0.001**
d-Threitol	0.02 [0.02-0.03]	0.03 [0.03-0.04]	**<0.001**
Methionine	1.91 [1.77-2.09]	1.61 [1.29-1.98]	**<0.001**
Oxoproline	50.23 [42.98-56.01]	61.32 [49.51-69.37]	**<0.001**
4-Hydroxyproline	1.75 [1.26-2.67]	1.71 [1.27-2.33]	0.836
Threonic acid	1.66 [1.51-1.94]	2.40 [1.79-3.55]	**<0.001**
Erythronic acid	0.06 [0.05-0.09]	0.11 [0.08-0.22]	**<0.001**
DL-2-Hydroxyglutaric acid	0.05 [0.04-0.05]	0.06 [0.05-0.08]	**<0.001**
a-ketoglutaric acid	0.80 [0.69-0.93]	0.73 [0.59-0.87]	**0.026**
Glutamic acid	3.59 [2.73-4.86]	8.07 [5.55-10.34]	**<0.001**
4-Hydroxybenzoic acid	0.02 [0.02-0.03]	0.03 [0.03-0.05]	**<0.001**
Phenylalanine	6.81 [6.43-7.27]	6.60 [5.56-7.72]	0.285
Dodecanoic acid	0.22 [0.16-0.29]	0.23 [0.17-0.33]	0.284
d-Xylose	0.03 [0.03-0.06]	0.05 [0.04-0.08]	**<0.001**
Taurine	0.62 [0.45-0.82]	1.33 [0.91-1.73]	**<0.001**
d-Arabinose	0.08 [0.06-0.09]	0.14 [0.11-0.23]	**<0.001**

d-Xylitol	0.00 [0.00-0.00]	0.00 [0.00-0.00]	**<0.001**
d-Arabitol	0.02 [0.01-0.02]	0.02 [0.02-0.03]	**<0.001**
Glycerol-1-phosphate	0.06 [0.06-0.08]	0.07 [0.05-0.09]	0.380
Glutamine	41.02 [35.52-50.56]	27.16 [14.54-38.64]	**<0.001**
Xylonic acid	0.14 [0.10-0.18]	0.05 [0.04-0.11]	**<0.001**
Ribonic acid	0.01 [0.01-0.01]	0.02 [0.01-0.04]	**<0.001**
3-Phosphoglyceric acid	0.01 [0.01-0.01]	0.03 [0.02-0.05]	**<0.001**
Ornithine	21.03 [16.59-24.92]	16.81 [13.13-23.86]	**0.009**
Citric acid	87.69 [68.20-110.80]	86.26 [58.21-125.49]	0.867
Tetradecanoic acid	1.03 [0.81-1.34]	1.03 [0.72-1.37]	0.537
Hippuric acid	1.01 [0.71-2.20]	0.91 [0.30-1.89]	0.275
Vanillylmandelic acid	0.02 [0.01-0.02]	0.03 [0.02-0.04]	**<0.001**
4-hydroxyPhenyllactic acid	2.16 [1.67-2.56]	2.36 [1.86-3.23]	0.229
d-Fructose	0.03 [0.03-0.04]	0.05 [0.03-0.14]	**<0.001**
d-Mannitol	0.03 [0.02-0.06]	0.14 [0.07-0.51]	**<0.001**
d-Mannonic acid	0.07 [0.05-0.10]	0.16 [0.11-0.30]	**<0.001**
d-Galactitol	0.02 [0.02-0.03]	0.02 [0.02-0.03]	0.519
Galacturonic acid	0.05 [0.04-0.06]	0.07 [0.05-0.12]	**<0.001**
Galactonic acid	0.00 [0.00-0.01]	0.01 [0.00-0.02]	**<0.001**
Saccharic acid	0.04 [0.03-0.07]	0.08 [0.05-0.13]	**<0.001**
Indole-3-propanoic acid	0.09 [0.05-0.17]	0.06 [0.03-0.09]	**0.001**
Myo-Inositol	0.64 [0.55-0.70]	0.89 [0.71-1.08]	**<0.001**
Uric acid	12.86 [7.95-16.96]	12.55 [7.92-16.87]	0.974
Sedoheptulose	0.05 [0.04-0.06]	0.07 [0.05-0.09]	**<0.001**
Indolelactic acid	0.32 [0.28-0.47]	0.50 [0.39-0.61]	**<0.001**
Linoleic acid	1.83 [1.39-3.40]	1.39 [0.86-2.59]	**0.008**
Oleic acid	11.46 [8.53-16.61]	9.83 [5.38-17.30]	0.096
Glucose 6-phosphate	0.00 [0.00-0.00]	0.01 [0.01-0.01]	**<0.001**
d-Sucrose	0.02 [0.01-0.03]	0.07 [0.04-0.15]	**<0.001**
Maltose	0.01 [0.01-0.01]	0.12 [0.05-0.22]	**<0.001**
a-Tocopherol	0.44 [0.31-0.56]	0.42 [0.27-0.60]	0.574

Results are presented in Relative Units (RU) and displayed as medians [interquartile ranges]. Statistical differences were assessed using the Mann-Whitney U test on the $\log_{10}$ RU.

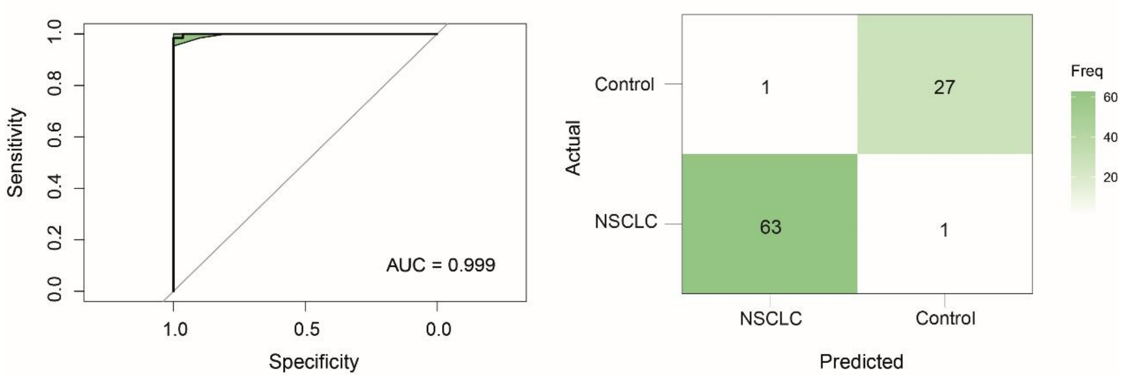

Figure S1. Receiver Operating Characteristic (ROC) curve and confusion matrix generated using the training set of the data and a 5-fold cross validated Support Vector Machine model based on the 8 selected metabolites. The model distinguishes between non-small cell lung cancer patients (NSCLC) and the control group. AUC: Area under the curve.

Table S2. Plasma metabolite profiles of non-small cell lung cancer (NSCLC) patients pre- and post-SBRT.

Plasma metabolites	Pre - SBRT<br>*n* = 45	Post - SBRT<br>*n* = 45	p-value
Pyruvic acid	6.18 [4.91, 7.79]	4.83 [3.56, 7.19]	0.052
Lactic acid	14.61 [12.94, 17.27]	13.68 [11.86, 15.70]	**0.028**
2-Hydroxyisobutyric acid	0.45 [0.37, 0.64]	0.52 [0.37, 0.71]	0.495
Glycolic acid	0.20 [0.17, 0.22]	0.18 [0.16, 0.22]	0.297
Alanine	2.94 [2.62, 3.56]	2.87 [2.56, 3.53]	0.346
2-Hydroxybutyric acid	3.98 [2.88, 5.72]	3.75 [2.36, 4.89]	0.105
3-methyl-2-oxobutyric acid	0.11 [0.09, 0.14]	0.11 [0.09, 0.15]	0.947
3-hydroxybutyric acid/3-hydroxyisobutyric acid	2.07 [0.99, 6.44]	2.11 [1.26, 3.99]	0.687
2-Hydroxyisovaleric acid	1.57 [1.13, 2.58]	1.62 [1.06, 2.17]	0.200
2-keto-3-methylvaleric acid	0.21 [0.17, 0.27]	0.20 [0.16, 0.26]	0.395
3-Hydroxyisovaleric acid	0.30 [0.24, 0.34]	0.29 [0.22, 0.33]	0.313
Valine	17.68 [15.62, 20.25]	18.20 [15.55, 20.60]	1.000
Benzoic acid	0.30 [0.22, 0.38]	0.26 [0.20, 0.37]	0.341
Ethanolamine	0.30 [0.25, 0.35]	0.29 [0.27, 0.35]	0.576
Leucine	9.39 [7.90, 11.13]	9.13 [8.13, 10.79]	0.553
Phosphoric acid	3.62 [2.78, 4.12]	3.88 [3.04, 4.32]	0.440
Glycerol	0.94 [0.68, 1.31]	0.85 [0.70, 1.24]	0.754
Ethylmalonic acid	1.16 [0.79, 1.42]	1.01 [0.84, 1.20]	0.297
Isoleucine	5.16 [3.95, 6.09]	4.95 [4.18, 6.21]	0.938
Proline	23.07 [13.62, 28.29]	19.91 [12.78, 29.03]	0.788
Glycine	1.31 [1.17, 1.40]	1.29 [1.24, 1.37]	0.257
Succinic acid	0.10 [0.09, 0.14]	0.10 [0.08, 0.13]	0.061
Glyceric acid	0.66 [0.55, 0.89]	0.65 [0.54, 0.79]	0.584
Fumaric acid	0.48 [0.22, 0.72]	0.37 [0.24, 0.57]	0.440
Serine	5.82 [4.41, 6.94]	5.95 [5.18, 7.32]	0.531
Threonine	6.75 [5.98, 8.00]	7.37 [6.11, 8.62]	0.143
Hydrocinnamic acid	0.12 [0.07, 0.17]	0.11 [0.08, 0.16]	0.858
Malic acid	0.14 [0.10, 0.19]	0.13 [0.11, 0.16]	**0.048**
d-Threitol	0.04 [0.03, 0.06]	0.03 [0.03, 0.05]	**0.022**
Methionine	1.66 [1.44, 2.17]	1.72 [1.54, 2.03]	0.252
Oxoproline	62.55 [50.28, 69.05]	52.12 [47.24, 70.23]	0.163
4-Hydroxyproline	1.91 [1.58, 2.68]	2.09 [1.45, 3.21]	0.335
Threonic acid	2.40 [1.84, 4.07]	2.37 [1.97, 3.53]	1.000
Erythronic acid	0.13 [0.09, 0.26]	0.15 [0.09, 0.22]	0.771
DL-2-Hydroxyglutaric acid	0.06 [0.05, 0.08]	0.06 [0.05, 0.07]	0.639
a-ketoglutaric acid	0.75 [0.59, 0.87]	0.68 [0.55, 0.87]	0.084
Glutamic acid	5.94 [4.29, 7.36]	5.33 [4.12, 6.95]	0.185
4-Hydroxybenzoic acid	0.03 [0.03, 0.04]	0.03 [0.02, 0.04]	0.069
Phenylalanine	6.72 [5.89, 8.17]	6.82 [5.92, 7.49]	0.453
Dodecanoic acid	0.25 [0.16, 0.34]	0.22 [0.17, 0.31]	0.849
d-Xylose	0.06 [0.04, 0.09]	0.05 [0.03, 0.07]	0.181
Taurine	1.10 [0.77, 1.52]	0.92 [0.69, 1.25]	**0.009**
d-Arabinose	0.15 [0.10, 0.24]	0.12 [0.09, 0.17]	**0.006**
d-Xylitol	0.00 [0.00, 0.00]	0.00 [0.00, 0.00]	**0.045**

d-Arabitol	0.02 [0.02, 0.03]	0.02 [0.02, 0.04]	0.823
Glycerol-1-phosphate	0.07 [0.06, 0.09]	0.08 [0.06, 0.09]	0.071
Glutamine	37.53 [23.67, 43.96]	35.99 [24.35, 43.01]	0.128
Xylonic acid	0.08 [0.05, 0.12]	0.09 [0.05, 0.12]	0.341
Ribonic acid	0.02 [0.01, 0.05]	0.02 [0.02, 0.04]	0.866
3-Phosphoglyceric acid	0.03 [0.02, 0.05]	0.02 [0.02, 0.03]	**0.014**
Ornithine	18.54 [16.25, 25.32]	17.31 [13.80, 22.48]	**0.007**
Citric acid	100.70 [68.09, 138.61]	85.88 [69.23, 136.88]	0.488
Tetradecanoic acid	1.03 [0.74, 1.45]	0.94 [0.77, 1.33]	0.663
Hippuric acid	1.05 [0.61, 2.12]	1.35 [0.50, 2.67]	0.297
Vanillylmandelic acid	0.03 [0.02, 0.05]	0.03 [0.02, 0.04]	0.938
4-hydroxyPhenyllactic acid	2.41 [1.93, 3.76]	2.42 [1.72, 3.31]	0.352
d-Fructose	0.04 [0.03, 0.09]	0.03 [0.03, 0.08]	0.225
d-Mannitol	0.16 [0.09, 0.55]	0.16 [0.07, 0.59]	0.884
d-Mannonic acid	0.17 [0.10, 0.28]	0.17 [0.11, 0.30]	0.704
d-Galactitol	0.02 [0.02, 0.03]	0.02 [0.01, 0.03]	0.192
Galacturonic acid	0.08 [0.05, 0.11]	0.07 [0.06, 0.12]	0.893
Galactonic acid	0.01 [0.00, 0.02]	0.01 [0.00, 0.02]	0.623
Saccharic acid	0.10 [0.06, 0.17]	0.10 [0.07, 0.18]	0.248
Indole-3-propanoic acid	0.06 [0.03, 0.10]	0.06 [0.04, 0.09]	0.407
Myo-Inositol	0.89 [0.73, 1.19]	0.79 [0.61, 1.14]	0.276
Uric acid	12.55 [8.16, 15.79]	11.77 [10.17, 15.28]	0.576
Sedoheptulose	0.08 [0.06, 0.09]	0.07 [0.04, 0.10]	**0.024**
Indolelactic acid	0.53 [0.45, 0.68]	0.49 [0.38, 0.74]	0.540
Linoleic acid	1.68 [1.09, 2.84]	1.91 [1.18, 2.70]	0.427
Oleic acid	11.53 [5.67, 20.14]	12.17 [7.40, 17.05]	0.553
Glucose 6-phosphate	0.01 [0.00, 0.01]	0.01 [0.00, 0.01]	0.240
d-Sucrose	0.07 [0.02, 0.17]	0.05 [0.02, 0.13]	0.313
Maltose	0.05 [0.03, 0.12]	0.04 [0.02, 0.07]	**0.003**
a-Tocopherol	0.43 [0.26, 0.62]	0.48 [0.29, 0.69]	**0.029**

Results are presented in Relative Units (RU) and displayed as medians [interquartile ranges]. Statistical differences were assessed using the Wilcoxon signed rank test on the log10 RU. SBRT: Stereotactic Ablative Radiation Therapy.

Table S3. Plasma metabolite profiles of non-small cell lung cancer (NSCLC) patients pre- and post-CFRT.

Plasma metabolites	Pre - CFRT<br>*n* = 23	Post -CFRT<br>*n* = 23	p-value
Pyruvic acid	8.02 [5.22, 10.28]	6.44 [5.39, 8.70]	0.709
Lactic acid	13.87 [12.53, 18.87]	15.75 [14.12, 19.51]	0.520
2-Hydroxyisobutyric acid	0.38 [0.35, 0.49]	0.43 [0.32, 0.52]	0.393
Glycolic acid	0.19 [0.16, 0.23]	0.18 [0.17, 0.23]	0.560
Alanine	3.02 [2.52, 3.37]	3.52 [3.08, 3.68]	0.111
2-Hydroxybutyric acid	4.02 [2.58, 5.54]	3.89 [3.05, 5.15]	0.754
3-methyl-2-oxobutyric acid	0.11 [0.09, 0.14]	0.11 [0.08, 0.13]	0.893
3-hydroxybutyric acid/3-hydroxyisobutyric acid	1.41 [0.85, 2.72]	1.71 [0.80, 2.56]	0.731
2-Hydroxyisovaleric acid	1.17 [1.06, 2.32]	1.44 [1.03, 1.80]	0.377
2-keto-3-methylvaleric acid	0.25 [0.16, 0.29]	0.25 [0.20, 0.34]	0.119
3-Hydroxyisovaleric acid	0.24 [0.20, 0.30]	0.25 [0.22, 0.30]	0.800
Valine	16.11 [13.51, 20.49]	17.48 [14.35, 19.98]	0.540
Benzoic acid	0.21 [0.18, 0.25]	0.19 [0.16, 0.23]	0.377
Ethanolamine	0.27 [0.25, 0.32]	0.27 [0.24, 0.32]	0.329
Leucine	7.44 [6.60, 11.02]	8.75 [7.78, 11.34]	0.126
Phosphoric acid	4.11 [3.01, 4.79]	4.42 [3.52, 5.27]	0.111
Glycerol	0.51 [0.38, 0.81]	0.51 [0.37, 0.84]	0.893
Ethylmalonic acid	0.92 [0.69, 1.20]	0.87 [0.71, 1.24]	0.917
Isoleucine	4.25 [3.88, 5.68]	5.16 [4.77, 6.49]	**0.023**
Proline	30.04 [23.39, 34.46]	32.01 [23.99, 39.55]	0.520
Glycine	1.19 [1.12, 1.32]	1.30 [1.20, 1.42]	**0.004**
Succinic acid	0.10 [0.08, 0.11]	0.09 [0.08, 0.11]	0.445
Glyceric acid	0.78 [0.52, 1.03]	0.65 [0.54, 0.89]	0.988
Fumaric acid	0.46 [0.30, 0.58]	0.57 [0.39, 0.94]	**0.045**
Serine	6.57 [5.00, 7.48]	7.30 [6.24, 8.29]	**0.007**
Threonine	6.71 [5.48, 7.60]	7.30 [6.47, 8.93]	0.061
Hydrocinnamic acid	0.09 [0.04, 0.17]	0.08 [0.05, 0.11]	0.377
Malic acid	0.11 [0.09, 0.15]	0.12 [0.08, 0.17]	0.964
d-Threitol	0.03 [0.03, 0.04]	0.03 [0.02, 0.04]	0.463
Methionine	1.45 [1.26, 1.70]	1.77 [1.45, 2.05]	**0.011**
Oxoproline	53.20 [45.73, 65.12]	49.38 [44.43, 59.26]	0.427
4-Hydroxyproline	1.60 [1.18, 1.80]	1.89 [1.27, 2.87]	0.151
Threonic acid	2.64 [2.16, 3.13]	2.96 [2.23, 3.78]	0.179
Erythronic acid	0.07 [0.06, 0.09]	0.07 [0.06, 0.09]	0.709
DL-2-Hydroxyglutaric acid	0.05 [0.04, 0.06]	0.05 [0.04, 0.06]	0.360
a-ketoglutaric acid	0.73 [0.62, 0.93]	0.86 [0.78, 1.07]	**0.048**
Glutamic acid	9.80 [8.85, 11.61]	9.64 [7.46, 13.22]	0.463
4-Hydroxybenzoic acid	0.04 [0.03, 0.05]	0.03 [0.02, 0.04]	0.134
Phenylalanine	6.48 [5.41, 7.38]	6.85 [5.91, 7.99]	0.080
Dodecanoic acid	0.23 [0.18, 0.26]	0.19 [0.16, 0.26]	0.540
d-Xylose	0.04 [0.03, 0.06]	0.04 [0.03, 0.06]	0.974
Taurine	1.49 [1.12, 1.69]	1.27 [0.85, 1.63]	0.111
d-Arabinose	0.15 [0.11, 0.18]	0.12 [0.08, 0.19]	0.160
d-Xylitol	0.00 [0.00, 0.00]	0.00 [0.00, 0.00]	0.465

d-Arabitol	0.02 [0.02, 0.03]	0.02 [0.02, 0.03]	0.893
Glycerol-1-phosphate	0.06 [0.05, 0.07]	0.06 [0.05, 0.07]	0.501
Glutamine	23.29 [14.34, 28.37]	27.46 [17.09, 36.59]	0.151
Xylonic acid	0.03 [0.03, 0.05]	0.04 [0.03, 0.05]	0.709
Ribonic acid	0.01 [0.01, 0.02]	0.01 [0.01, 0.02]	0.823
3-Phosphoglyceric acid	0.04 [0.03, 0.05]	0.04 [0.03, 0.06]	0.284
Ornithine	14.43 [12.52, 17.51]	17.37 [13.03, 22.16]	0.427
Citric acid	97.40 [68.36, 123.65]	105.55 [70.49, 148.37]	0.234
Tetradecanoic acid	1.00 [0.81, 1.12]	0.79 [0.74, 1.17]	0.731
Hippuric acid	0.81 [0.12, 1.40]	0.62 [0.06, 1.58]	0.754
Vanillylmandelic acid	0.02 [0.02, 0.03]	0.02 [0.02, 0.03]	0.687
4-hydroxyPhenyllactic acid	2.28 [1.86, 2.87]	2.22 [1.90, 3.15]	0.223
d-Fructose	0.05 [0.04, 0.17]	0.05 [0.03, 0.12]	0.871
d-Mannitol	0.08 [0.05, 0.30]	0.05 [0.04, 0.08]	0.065
d-Mannonic acid	0.12 [0.10, 0.20]	0.11 [0.08, 0.23]	0.643
d-Galactitol	0.02 [0.02, 0.03]	0.03 [0.02, 0.03]	0.673
Galacturonic acid	0.07 [0.06, 0.09]	0.06 [0.04, 0.08]	0.329
Galactonic acid	0.01 [0.00, 0.02]	0.01 [0.00, 0.01]	0.665
Saccharic acid	0.06 [0.04, 0.08]	0.05 [0.04, 0.07]	0.345
Indole-3-propanoic acid	0.05 [0.03, 0.09]	0.05 [0.04, 0.09]	0.846
Myo-Inositol	0.95 [0.69, 1.06]	0.83 [0.68, 1.02]	0.665
Uric acid	9.96 [6.60, 19.11]	8.98 [4.79, 20.50]	0.520
Sedoheptulose	0.05 [0.04, 0.07]	0.05 [0.04, 0.06]	0.200
Indolelactic acid	0.46 [0.32, 0.62]	0.37 [0.29, 0.47]	0.061
Linoleic acid	0.93 [0.63, 1.55]	1.04 [0.69, 1.96]	0.731
Oleic acid	8.05 [4.04, 11.33]	9.58 [4.69, 14.85]	0.777
Glucose 6-phosphate	0.01 [0.01, 0.01]	0.01 [0.01, 0.01]	0.846
d-Sucrose	0.09 [0.05, 0.13]	0.08 [0.04, 0.13]	0.870
Maltose	0.22 [0.13, 0.24]	0.13 [0.10, 0.19]	**0.028**
a-Tocopherol	0.39 [0.27, 0.43]	0.40 [0.24, 0.60]	0.893

Results are presented in Relative Units (RU) and displayed as medians [interquartile ranges]. Statistical differences were assessed using the Wilcoxon signed rank test on the log10 RU. CFRT: Conventionally Fractionated Radiation Therapy.
